# Clinical aspects of mCRPC management in patients treated with radium-223

**DOI:** 10.1038/s41598-020-63302-2

**Published:** 2020-04-21

**Authors:** Elisa Lodi Rizzini, Valeria Dionisi, Pietro Ghedini, Alessio Giuseppe Morganti, Stefano Fanti, Fabio Monari

**Affiliations:** 1 Nuclear Medicine, S. Orsola-Malpighi Hospital, Bologna, Italy; 2 DIMES University of Bologna, Bologna, Italy; 3 Radiation Oncology Center, S. Orsola-Malpighi Hospital, Bologna, Italy

**Keywords:** Radiotherapy, Prostate cancer

## Abstract

Bone is the most common site of metastasis in metastatic castration-resistant prostate cancer (mCRPC), which is associated with pain and skeletal events. Radium-223 dichloride (Xofigo) is an alpha-emitting radioactive isotope that can specifically target bone lesions. Herein, we report the results of a retrospective analysis that documents our experience in the use of radium-223. Data from 63 patients (pts) with mCRPC who underwent radium-223 treatment from December 2015 to September 2017 were collected. Radium-223 (55 kBq/kg) was administered every 4 weeks for up to 6 cycles. The primary endpoint was OS. Radium-223 was administered as first line therapy in 11 pts, as second line in 19 pts, as third line in 16 pts and in successive lines in 17 pts; 42 pts out of 63 (67%) completed all six cycles. Within one month after the end of 6 cycles of radium-223, 15 pts out of 42 (35.7%) had achieved PR, 11 pts out of 42 (26.2%) had SD and 14 pts out of 42 (33.3%) had PD. Levels of pain decreased with progressive cycles of radium-223. After a minimum follow-up of 2 months and a maximum of 43 months, median OS was 15 months and median PFS was 8 months. The most frequent radium-223 related toxicity was low grade haematologic toxicity, predominantly G1-G2, that occurred halfway through treatment in about 75% of pts. The favourable results reported herein confirm that radium-223 can be considered well tolerated and effective in mCRPC, and is associated with significant decreases in pain.

## Introduction

Prostate cancer is one of the most commonly diagnosed malignancies and a leading cause of cancer death in men^1^. In 2012, there were over 1,000,000 newly diagnosed cases and >307,000 deaths from the disease worldwide^1^. In Europe, in 2018, there were an estimated 450,000 new cases of prostate cancer^2^, and in the US, prostate cancer is the second leading cause of cancer deaths following those of the lung and bronchus^3^. Even if several treatments are local, including radical prostatectomy, external radiotherapy and brachytherapy, which may allow eradication of disease in some patients (pts), roughly one-third of men will develop distant metastases^4^. Even if long-term survival is relatively high, averaging 10 years in those with localised disease, in men with metastatic disease the 5-year survival rate decreases dramatically to approximately 30%^5^. Such poor survival rates can be attributed in large part to resistance to treatment in which aggressive variants of prostate cancer arise following the accumulation of mutations in key tumour suppressor genes^6,7^. Moreover, androgen depletion is known to induce genes involved in cancer progression and metastasis and the epithelial-to mesenchymal transition, which have implicated the bone-epithelial interaction as a key player in the progression of prostate cancer^8^. Bone metastases dominate the clinical picture of advanced prostate cancer and are a major source of morbidity in metastatic disease^9^. In pts with recurrence, bone is, in fact, the most common site of metastasis affecting more than 90% of men with metastatic castration-resistant prostate cancer (mCRPC)^10^, which is associated with pain and skeletal events such as fracture and spinal cord compression that clearly decrease the quality of life^11^.

Radium-223 dichloride (Xofigo) is an alpha-emitting radioactive isotope that induces irreversible DNA double-strand breaks and leads to death of tumour cells^11^. As a calcium-mimetic, radium-223 is considered as ‘bone-seeking’ and can specifically target bone lesions. Based on the results of the ALSYMPCA study, radium-223 is now considered as a valid and efficient therapy for mCRPC with bone lesions, in combination with systemic therapies. ALSYMPCA was a phase 3, double-blind, placebo-controlled study in which 921 pts with mCRPC were randomised to receive six injections of radium-223 (at a dose of 50 kBq/kg) or placebo every 4 weeks, in addition to best standard of care^10^. Considering the primary endpoint of overall survival (OS), radium-223 showed a significant improvement over placebo (median, 14.9 months vs. 11.3 months, respectively), or a 30% reduction in the risk of death. All main secondary efficacy endpoints also showed a benefit of radium-233 over placebo. In addition, significantly more pts receiving radium-223 had a meaningful improvement in the quality of life. Importantly, the overall incidence of adverse events was also lower in the radium-223 group vs. placebo for all grades.

Another important study in mCRPC with radium-223 is ERA 223 which was carried out in men with asymptomatic or mildly symptomatic chemotherapy-naïve pts with bone-predominant mCRPC who were randomised to radium-223 in combination with abiraterone acetate and prednisone (AAP) or matching placebo^12^. A higher rate of fractures was seen in the group who received radium-223, which had a fracture rate of 26% compared with 10% for those receiving abiraterone and prednisone alone. The primary endpoint of the trial was symptomatic skeletal event-free survival (SSE-FS) rate, which was similar for both groups (49% for radium-223 and AAP vs 47% for AAP plus placebo). Median SSE-FS was 22.3 months with the combination and 26.0 months for AAP alone. Median OS was 30.7 months with the combination and 33.3 months with AAP alone, and the difference of 2.6 months was not significant. Based on this consideration, in the EU, radium-223 is currently approved for monotherapy or in combination with a luteinising hormone-releasing hormone (LHRH) analogue for the treatment of adult pts with mCRPC, symptomatic bone metastases and no known visceral metastases, in progression after at least two prior lines of systemic therapy for mCRPC (other than LHRH analogues), or ineligible for any available systemic mCRPC treatment. In this regard, the authors of a recently published letter noted their disagreement with the EMA Pharmacovigilance Risk Assessment Committee (PRAC) interpretation of the available data from both ERA 223 and ALSYMPCA, agreeing that radium-223 should not be used in combination with abiraterone acetate and prednisolone/prednisone, but differing with the recommendation that it should be reserved for third or later lines of therapy in mCRPC as this may restrict access to many pts who could potentially benefit from it^13^. Of note, other regulatory agencies including the FDA, the Canadian Agency for Drugs and Technologies in Health, and the Japanese Pharmaceuticals and Medical Devices Agency have assessed the same data and decided on no change to the label for radium-223^13^.

In our institution, radium-223 is considered as a valid therapeutic for mCRPC pts with symptomatic bone metastases and without visceral involvement. We are currently gaining more experience with radium-223 in this patient group, and to date have treated almost 80 pts. Herein, we report the results of a retrospective analysis that shares our experience in the use of radium-223 to manage pts with mCRPC.

## Methods

### Study design

Data from pts with mCRPC who underwent radium-223 treatment from December 2015 to September 2017 at Sant’Orsola-Malpighi University were collected retrospectively. Data were collected using a standardised case report form that includes patient age, weight, height, comorbidities, concomitant therapies, cancer history, lines of therapy prior to receiving radium-223, relevant outcomes, therapies administered for pain, supportive therapy and adverse events correlated with treatment. Radium-223 (55 kBq/kg) was administered every 4 weeks for up to 6 cycles. The primary endpoint was OS. Secondary endpoints included: progression-free survival (PFS), response based on biomarkers (prostate serum antigen [PSA] and alkaline phosphatase [ALP]), pain evaluation using numeric rating scale (NRS), safety, response rate according to clinical and radiologic criteria (whole-body scan [WBS], computed tomography [CT] and/or positron emission tomography [PET]) and evaluation of adequate supportive therapy allowing for completion of all six administrations of radium-223.

### Patient evaluations

For each patient, the number of bone lesions was assessed by a bone WBS using Tc-99 before starting treatment. At one month after the end of all radium-223 administrations, a bone WBS with Tc-99 was performed to assess the response to treatment. Prior to monthly injections, pts were evaluated with clinical exam (ECOG score and NRS scale) and laboratory exams including complete blood count (CBC), PSA and ALP. Toxicity during radium-223 therapy was evaluated with Common Criteria for Adverse event (CTACAE) scale ver. 4.3. Pts were also followed at 3 and 6 months after the last injection of radium-223 with clinical, laboratory and radiological evaluation with ^11^C-choline PET/CT. In pts who were deemed fit to receive bisphosphonate therapy, zoledronic acid (4 mg iv monthly) was administered during treatment with radium-223. Mean and median survival and cumulative death incidence were also evaluated.

According to MDA cancer response criteria^14^, response to radium-223 was classified by bone WBS after 1 month to the end of therapy as partial response (PR), stable disease (SD), or progressive disease (PD) on the basis of the bone WBS in association with PSA and ALP over time; PR was defined as ≥50% subjective decrease in tracer uptake, SD was defined as no substantial changes in tracer uptake of known lesions and PD was defined as ≥25% subjective increase of tracer uptake or the appearance of new tracer uptake. In some cases before the beginning of the treatment, chest-abdominal CT was performed to assess the absence of visceral metastasis.

An ^11^C-choline PET/CT scan was performed after 3 and 6 months after the end of radium-223 therapy for disease restaging; according to the same assessment response criteria of bone WBS, with the help of an expert nuclear medicine physician, we defined ^11^C-choline PET/CT disease restaging as PR, SD and PD. PD and PFS were also assessed by the time to the first skeletal event (such as bone pain or symptomatic pathological bone fractures treated with external beam radiation therapy and/or orthopaedic surgical intervention). Response disease was assessed by monitoring the trends in ALP and PSA over time during treatment and follow-up, searching for possible associations between their levels and clinical data; compared to imaging, in the presence of decreasing trend of PSA and ALP values, there was SD/PR, while a growing trend of PSA and ALP values indicated PD. No PSA or ALP cuts-off were taken into consideration to assess disease response.

### Inclusion and exclusion criteria

We retrospectively analysed the clinical course and treatment response of the first 63 pts who received radium-223 therapy. Eligible criteria for treatment were hormone-refractory prostate cancer, presence of two or more symptomatic bone metastases, no known visceral metastases and Eastern Cooperative Oncology Group (ECOG) performance status score of 0–2. These pts could have been previously subjected to any systemic therapy line such as taxanes, abiraterone, or enzalutamide. Pts were excluded if they received chemotherapy within the previous 4 weeks, were asymptomatic, had a malignant lymphadenopathy that was >3 cm in the short-axis diameter, had haemoglobin <\10 gm/dl, a white blood cell count <1.5 × 10^9^ and a platelet count <100 × 10^9^ at the first cycle of radium-223. The study was approved by the local ethics committee of Bologna University Hospital St. Orsola-Malpighi (Comitato Etico Indipendente di Area Vasta Emilia Centro della Regione Emilia-Romagna - CE-AVEC). All data were collected according to the national and international regulations for clinical trial research. Written informed consent was obtained from all individual participants included in the study.

### Statistical analysis

Descriptive statistics were used for patient characteristics and clinical measures of interest. Data are presented as percentages, median and interquartile range (IQR) and means ± SD. The average change at each time for PSA, ALP and NRS was calculated using the paired-samples t-test. A logarithmic transformation was used to correct the non-normality of variables (PSA, ALP and NRS). Shapiro-Wilk test was also used to assess normality of continuous variables. Differences were considered significant at P < 0.05.

The number of bone lesions was compared with PSA and ALP levels at baseline, at the third and sixth radium-223 cycles, and at 1 and 3 months after the end of treatment; the number of prior systemic therapies was compared with ALP and PSA levels at different treatment times as well as with haemoglobin, NRS and anaemia. Comparisons between the levels of PSA, ALP, NRS and number of previous lines of therapy/anaemia were also evaluated. A 1-way ANOVA, followed by Bonferroni’s correction was used for all multiple comparison analysis tests. The Bonferroni adjustment multiplies each of the significance levels computed from the test by the number of comparisons (J * (J − 1)/2), where J is the number of groups. Hence, under Bonferroni, a new level of significance will be computed as *α*_*bonferroni*_ = 0.05 * (number of comparisons) and the results under this level will be considered significant. Effect size for each comparison was reported as η² with a 95% confidence interval (CI) and represents the proportion of variance in dependent variables explained by the independent variable (a η² > 0.14 was considered as a large effect size).

OS and PFS were calculated using the Kaplan-Meier estimation. Mean and median values were calculated. PFS and OS were defined as the time interval from the first administration of radium-223 to the earliest date of disease progression or death. We calculated OS in pts undergoing radium-223 at the first line after hormonal therapy, and at the second, third, fourth and fifth lines. We also compared individual and cumulative curves to each other (i.e. the first and second radium-223 lines vs the third, the fourth and fifth lines vs the third, the first, the second and the third curve vs the fourth, and the first, the second and the third curve vs the fifth). Differences in OS were evaluated using log rank test with statistical significance defined as P < 0.05. Effect size of comparison between curves was computed using hazard ratios (HR) with 95% CI estimated by a univariable Cox regression model. SPSS software (ver. 24.0) was used for statistical analyses.

### Ethics approval and consent to participate

This was a retrospective study and was approved by the local ethics committees. All data were collected according to the national and international regulatory for clinical trial research. Written informed consent was obtained from all individual participants included in the study.

## Results

### Patient disposition and baseline characteristics

From December 2015 to September 2017, we treated 63 pts affected by mCRPC with symptomatic bone metastases. Median age of the group was 76.8 years [IQR 71–79.5]. The main characteristics of the patient cohort are detailed in Table 1. More than half of pts suffered from a cardiological comorbidity and almost one-third had endocrine disease. Over half of pts had undergone radical surgery (prostatectomy), and 33% had received adjuvant radiotherapy.

**Table 1:** Baseline demographic, clinical, and pathological characteristics of the patient cohort.

Characteristic	Patients (n = 63)
Median age in years (IQR)	76.8 (71–79.5)
Prostatectomy, no (%)	35 (55.6)
Adjuvant radiotherapy, no (%)	21 (33.3)
Time since diagnosis of bone metastases (years), median (IQR)	2 (1–4)
***Concomitant disease***	
Cardiologic, no (%)	36 (57.1)
Endocrinology, no (%)	20 (31.7)
Cerebral, no (%)	5 (7.9)
Respiratory, no (%)	2 (3.2)
Neurologic/psychiatric, no (%)	3 (4.8)
Musculoskeletal, no (%)	4 (6.4)
Renal, no (%)	10 (15.9)
Other cancers, no (%)	7 (11.1)
Other, no (%)	6 (9.5)
Chemotherapy-naive, no (%)	11 (17.4)
1 Line of therapy before 223-Ra, n (%)	52 (82.5)
2 Lines of therapy before 223-Ra, n (%)	33 (52.4)
3 Lines of therapy before 223-Ra, n (%)	17 (27)
4 Lines of therapy before 223-Ra, n (%)	6 (9.5)
5 Lines of therapy before 223-Ra, n (%)	1(1.6%)
**Radium-223 treatment**	
*First-line*	11 (17.5)
*Second-line*	19 (30.5)
*Third-line*	16 (25.4)
*Fourth-line*	11(17.5)
*Fifth-line*	6 (9.5)
***ECOG performance status, no. (%)***	
0	46 (74.2)
0/1	5 (8.1)
1	5 (8.1)
2	6 (9.7)
***Bone scan lesions, no. (%)***	
<6	5 (7.9)
6–20	33 (52.4)
>20	16 (25.4)
Unknown	9 (14.3)
***Median biochemistry (IQR)***	
PSA prior to first 223Ra cycle, ng/mL	48.4 (17.8–131)
ALP prior to first 223Ra cycle, µg/L	138 (79–288)
Initial NRS pain score prior to first 223Ra cycle, median (IQR)	3 (2–5.5)
Hb levels prior to first 223-Ra cycle, g/dL, mean (DS)	12.2 (1.3)

Among our group of pts, the median time of developing mCRPC was 2 years [IQR 1–4] after the first metastases were diagnosed; 25% of pts developed mCRPC within 1 year, while 75% of pts developed mCRPC within 4 years. In this cohort, 52 pts out of 63 (82.5%) had undergone at least 1 line of systemic therapy before starting radium-223: the most commonly used drug was docetaxel (53.8%), while 33 pts out of 63 (52.4%) had undergone 2 lines of systemic therapy: abiraterone (50%) and cabazitaxel (18.8) were the most common drugs administered. Seventeen pts out of 63 had been treated with 3 lines of systemic therapy: abiraterone (38.9%), cabazitaxel (33.3%) and enzalutamide (16.7%). Only 6 pts out of 36 (9.5%) had undergone 4 lines of systemic therapy; 57.1% had been administered enzalutamide. Radium-223 was administered as first line therapy in 11 pts, as second line in 19 pts, as third line in 16 pts and in successive lines in 17 pts. The median number of cycles was 6 (range 3–6).

The number of bone lesions was evaluated by bone scan with Tc-99 before starting treatment; 5 pts out of 63 (7.9%) had fewer than 6 lesions and 16 pts out of 63 (25.4%) had more than 20 lesions, while 52.4% of pts had from 6 to 20 bone lesions. Performance status of the majority of pts who started radium-223 therapy was very good; 74.2% had an ECOG of 0 and only 6 out of 63 (9.7%) cases had an ECOG of 2 (Table 1).

### Radiologic assessment

Within one month after the end of 6 cycles of radium-223, on the basis of bone WBS, 15 pts out of 42 (35,7%) had achieved PR, 11 pts out of 42 (26,2%) had SD and 14 pts out of 42 (33,3%) had PD. No radiological assessment was done for 2 pts out of 42 because their clinical PD. On the basis of ^11^C-choline PET/CT scan and increased levels of PSA/ALP, at 3 months and 6 months, respectively, 24 pts out of 42 (57%) and 13 pts out of 42(31%) had PD, 4 pts out of 42 (9.5%) and 1 pt (2.4%) had PR, 8 pts out of 42 (19%) and 10 pts out of 42 (23.8%) had SD. At 6 months, 6 pts out of 42 (14.3%) at 3 months and 15 pts out of 42 (35.7%) were lost to follow-up or in clinical PD. At 6 months, 3 pts out of 42 were dead.

### PSA and ALP

**Figure 1:**
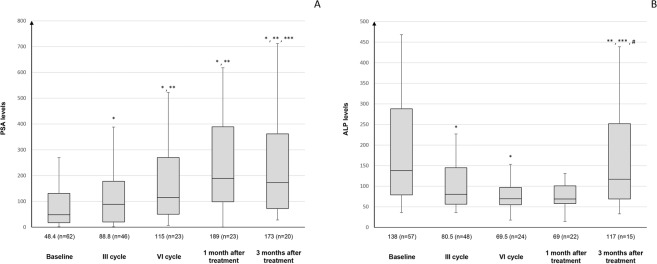
Levels of PSA (**A**) and ALP (**B**) at baseline, after the third and 6 cycles of radium-223, and at one and 3 months after treatment. Data are reported as median values and interquartile range. *p < 0.05 vs. baseline; **p < 0.05 vs after the 3rd cycle; ***p < 0.05 vs after the 6th cycle; ^#^p < 0.05 vs. one month.

In pts who started radionuclide therapy, PSA tended to increase, from a median baseline value of 48.4 ng/ml [IQR 17.8–131] to a median value that more than doubled after the sixth cycle (115 ng/ml [IQR 50.2–270]). During follow-up, PSA continued to increase, even at one month after the end of treatment (median 189 ng/ml [IQR 98.8–389]), with few exceptions. At 3 months after the last cycle radium-223, the levels of PSA began to decrease (Fig. 1A).

For ALP, the levels started to decline after the first three radium-223 cycles (from a median of 138 U/L [IQR 79–288] to 80.5 U/L [IQR 56.5–145]) and continued to decrease until the last cycle and at one month after the end of radionuclide therapy (69 U/L [IQR 58.0–101.0]). ALP values began to slightly increase at three months following the last cycle to a median value of 117 U/L [IQR 69–252] (Fig. 1B).

### Comparison between the levels of PSA and ALP and number of previous lines of chemotherapy

**Figure 2:**
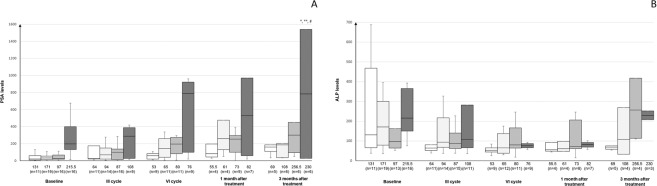
Changes in levels of PSA (**A**) and ALB (**B**) and number of lines of previous chemotherapy. Data are shown as median and interquartile ranges. *p < 0.05 vs. no previous chemotherapy; **p < 0.05 vs. 1 line; #<0.05 vs. 2 lines.

Only baseline PSA was significantly associated with the number of previous lines of chemotherapy lines (η² = 0.29, 95% CI = 0.08–0.44, p < 0.0004), and the group of pts who underwent more than one line of chemotherapy had a higher baseline PSA value. Median PSA in pts who had undergone one or more lines of chemotherapy for mCRPC doubled after the third cycle of radium-223 and started to decrease at month one after the end of radiotherapy. An exception to this was pts receiving three lines of chemotherapy in whom a greater increase in PSA was seen at the end of therapy compared to pts who had undergone one or two lines. In pts who were chemotherapy naïve, PSA levels from baseline to the third cycle of radium-223 increased less than in other pts. At three months after the end of radium-223, in all pts in our cohort, PSA began to increase, except for pts who had undergone only one line of chemotherapy (Fig. 2A).

On the other hand, there was no difference between ALP and number of lines of chemotherapy; there were no significant differences between any patient group, and ALP tended to increase over time (p = 0.33, η² = 0.07, 95% CI = 0.00–0.20 for I cycle; p = 0.32, η² = 0.08, 95% CI = 0.01–0.22 for III cycle; p = 0.43, η² = 0.13, 95% CI = 0.00–0.32 for VI cycle); the greatest increases were seen at one and three months after the end of therapy (Fig. 2B).

During treatment, pts who were deemed fit for bisphosphonate therapy were administered zoledronic acid, and the number of pts receiving this therapy increased from the first to the sixth cycle. However, there was no significant difference between levels of ALP level and administration of zoledronic acid (p = 0.94 for I cycle, p = 0.64 for II cycle, p = 0.88 for III cycle, p = 0.57 for IV cycle, p = 0.68 for V cycle and p = 0.44 for VI cycle).

### Comparison between levels of PSA and ALP and anaemia

**Figure 3:**
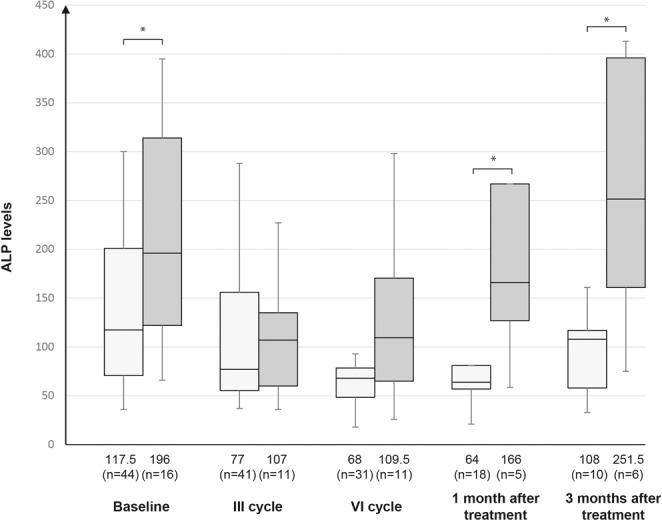
Levels of ALP and anaemia. Data are shown as median and interquartile range. *p < 0.05 anaemia vs. no anaemia.

There was no significant difference between the levels of PSA and anaemia during treatment with radium-223 (p = 0.73 for I cycle, p = 0.34 for III cycle and p = 0.55 for VI cycle). However, a significant difference between the levels of ALP and anaemia was seen at one month after the end of treatment (η² = 0.33, 95% CI = 0.04–0.56, p < 0.0052); anaemia was less common in pts with a decrease in ALP (from 166 U/L to 64 U/L) within one month after radium-223 compared to those with no decrease (Fig. 3).

### Comparison between ALP and haemoglobin levels and number of bone metastases

There was no difference between ALP levels at baseline (η² = 0.05, 95% CI = 0.00–0.19, p = 0.27) or one month follow-up (η² = 0.05, 95% CI = 0.00–0.25, p = 0.65) and number of bone lesions. However, there was a significant difference between baseline haemoglobin and the presence of >20 bone metastases (η² = 0.10, 95% CI = 0.00–0.29, p = 0.029).

### Pain

Levels of pain decreased with progressive cycles of radium-223. The median value of baseline pain as assessed by NRS was 3 [IQR 2–5.5], 2 [IQR 0–3.5] after the third cycle of radium-223 and 1 [IQR 0–3.5] at the end of radium-223 treatment. Regarding antalgic therapy, 12 pts out of 63 had stopped or reduced the dose of drugs for pain at the end of treatment, 8 pts out of 63 had introduced or increased the dose of pain medication, 10 pts out of 63 had never taken pain medications during therapy and 28 pts out of 63 did not change the type or dose of medication for pain. We could not evaluate changes in antalgic therapy for 5 pts out of 63 since they discontinued radium-223 therapy.

We found a significant difference between NRS levels and anaemia at the third cycle of radium-223 (η² = 0.10, 95% CI = 0.00–0.26, p = 0.0317); pts with anaemia had a lower NRS during the entire treatment period. We found no significant differences (η² = 0.04, 95% CI = 0.00–0.13, p = 0.5771) between NRS scores and number of prior lines of chemotherapy.

With cytoreductive/antalgic intent, 11 out of 63 pts treated with radium-223 received external beam radiotherapy with conventional fractionation aimed at reducing pain (8 Gy in a single dose, 20 Gy in 5 fractions or 30 Gy in 10 fractions) during therapy with radium-223 or in the following months. No pathologic fractures related to the disease occurred during follow-up.

### Survival

**Figure 4:**
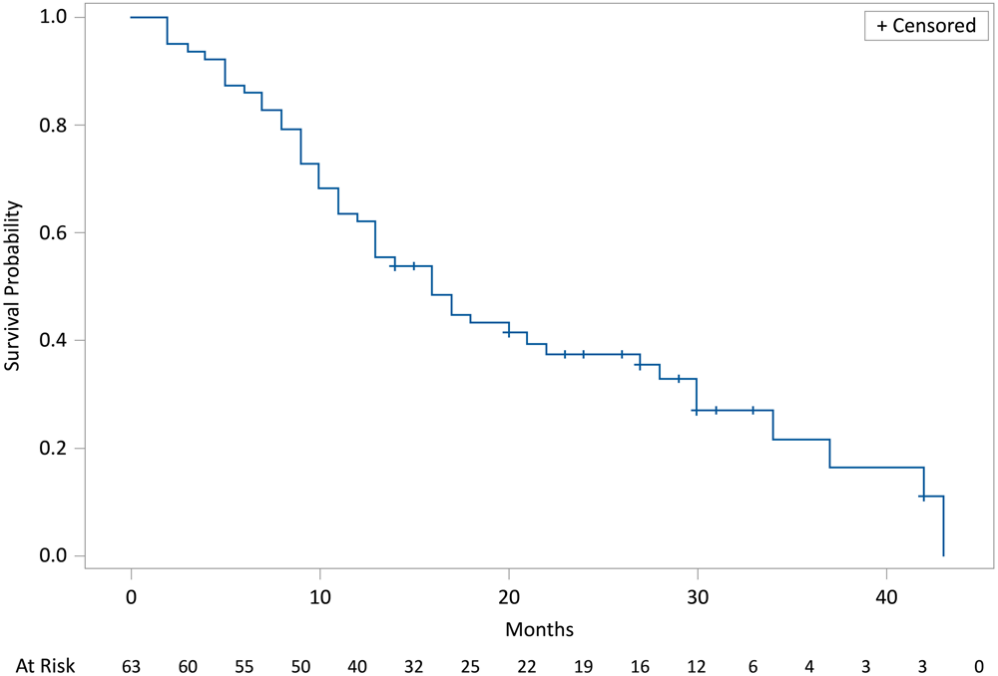
Kaplan-Meier estimates for OS.

**Figure 5:**
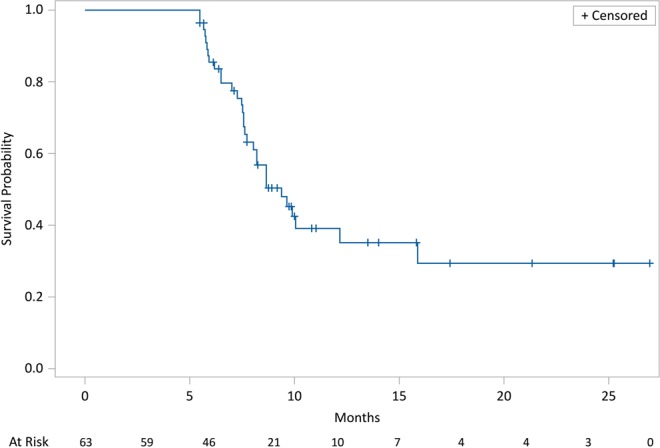
Kaplan-Meier estimates for PFS.

**Figure 6:**
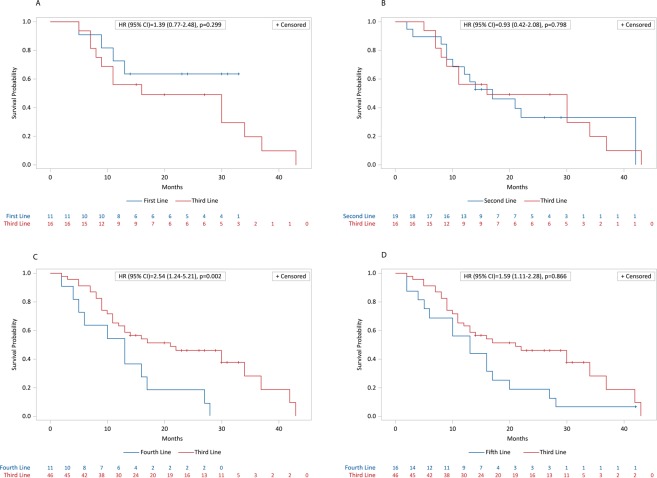
Kaplan-Meier estimates of OS for first vs. third lines of radium-223 (**A**), second vs third lines (**B**), third vs fourth Ra-223 lines (**C**), and third vs fifth lines (**D**).

**Figure 7:**
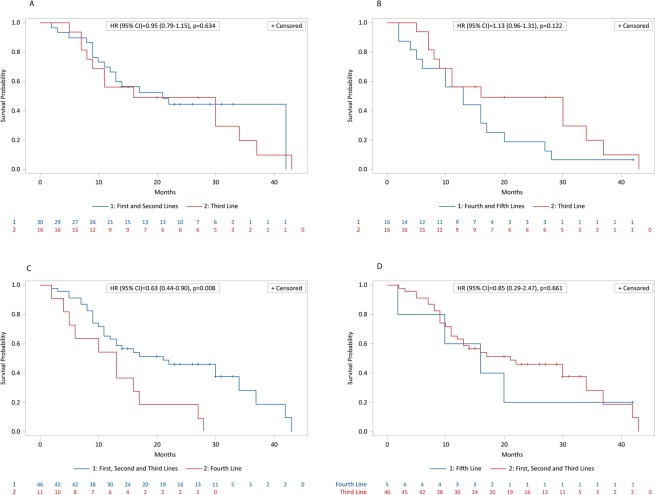
Kaplan-Meier estimates of OS. (**A**) First and second lines vs third line. (**B**) Third line vs. fourth and fifth lines. (**C)** First, second, and third lines vs fourth line. (**D**) First, second, and third lines vs fifth line.

After a minimum follow-up of 2 months and a maximum of 43 months, mean and median OS were, respectively, 17.5 months and 15 months (95% CI: 12.2–19.0); 17 pts out of 63 (27%) were alive, while 46 pts out of 63 (73%) had died. Kaplan-Meier survival estimates for median OS are shown in Fig. 4. Median PFS was 8 (95% CI: 7.0–8.7) months (46 progression or death, 17 with no progression). Kaplan-Meier survival estimates for PFS are shown in Fig. 5. Pts treated with radium-223 in first line had a mean and median OS respectively of 20,3 months and 23 months, while mean and median OS of pts treated with third line radium-223 line was 19.4 months and 15.5 months. The comparison between the different radium-223 lines was significant only between the third line and fourth lines (HR (95%IC) = 2.54 (1.24–5.21); log-rank test = 0.002) (Fig. 6C), and between the first, second, and third cumulative lines and the fourth line (HR (95% IC) = 0.63 (0.44–0.90); log-rank test = 0.008) (Fig. 7C).

#### Tolerability

All pts in our cohort underwent at least one administration of radium-223,19 pts out of 63 (30.2%) had to interrupt treatment due to toxicity and 2 pts out of 63 (3.2%) for PD; 42 pts out of 63 underwent all six monthly injections (67%). In addition, 9% of pts stopped treatment at the third Radium-223 administration. From the start of radium-223 therapy to the last follow-up, adverse events related to external beam radiotherapy (i.e. symptomatic lesions but not pathologic bone fractures) were observed in 11 pts out of 63 for a total of 12 events (one patient had two events).

The most frequent radium-223 related toxicity was low grade haematologic toxicity, especially anaemia, predominantly G1-G2, that occurred halfway through treatment in about 75% of pts, followed by leukopenia in 36% of pts and thrombocytopenia (21%) after the third cycle of radium-223. Myelosuppression was present at the sixth cycle of radium-223, where 25% of pts had a low level of haemoglobin, 6% of pts had leukopenia and about 3% of pts had thrombocytopenia.

The incidence of other symptoms such as asthenia, diarrhoea and nausea was low, seen, respectively, in 9%, 6% and 2% pts; these adverse effects were transient in most pts (data not available in 15 pts) and were no longer present after the last cycle of radium-223.

Anaemia was more common in pts who had previously underwent from two to three lines of chemotherapy (p = 0.27). There was a significant difference between baseline haemoglobin (12.24 ± 1.26) and post-therapy haemoglobin levels (10.72 ± 1.55; p < 0.001).

## Discussion

Adequate clinical management in pts undergoing treatment with radium-223 is of crucial importance to allow them to maintain a satisfying quality of life and continue all 6 cycles of therapy in order to obtain the maximum therapeutic benefit. Adequate management is also fundamental when considering the new indications for radium-223 in which it can be administered in pts with mCRPC, symptomatic bone metastases and no known visceral metastases, in progression after at least two prior lines of systemic therapy for mCRPC (other than LHRH analogues), or ineligible for any available systemic mCRPC treatment^15^. (Radium-223, Summary of Product Characteristics).

Based on our experience, the three main factors affecting treatment are early management of anaemia and levels of haemoglobin, prevention of adverse skeletal events and control of pain. In our analysis, anaemia was a sign of advanced disease more than as a result of therapy, since pts with a high number of bone metastases (>20) at the beginning and end of therapy had lower haemoglobin levels than pts with a smaller number of bone metastases at the beginning of therapy. Furthermore, anaemia seems to be related to the disease itself, and not to treatment, since pts with a decreasing trend of ALP levels had higher haemoglobin levels compared to pts with no ALP decrease during treatment. In our centre, management of anaemia has gradually changed based on our experience, and the first 30 pts were heavily pre-treated and not managed adequately. Statistical analysis of our data, in fact, showed a tendency towards increased NRS scores in pts with anaemia vs. those without anaemia, which reached significance halfway through therapy. Although we cannot rule out the contribution of radium-223 therapy to the increase in anaemia seen in our sample, we can say that pts with lower haemoglobin levels at the start of therapy because of a higher number of metastases, had a similar trend in haemoglobin levels during radium-223 treatment with an adequate supportive care for anaemia than pts with fewer metastases. This is in line with literature^16,17^ and the results from the ALSYMPCA trial in which anaemia as a serious adverse event was reported in 8% of pts with radium-223 and in 9% of pts treated with placebo^10^. It is also in agreement with real-world experience^18^, and in a study in 92 pts with mCRPC only baseline haemoglobin and ECOG PS were significantly correlated with OS among all clinical parameters^19^. For this reason, in our opinion, early identification of pts with sideropenic anaemia in which an oral iron supplement was sufficient to restore haemoglobin levels and improve anaemia is mandatory^20,21^. On the contrary, pts with an evident anaemic state but high values of iron, high transferrin saturation percentage (TSAT) and increased inflammatory indices (D-dimer, ferritin, fibrinogen, erythrocyte sedimentation rate and C-reactive protein), iron supplementation may not be of benefit. Unfortunately, TSAT and inflammatory markers were measured in only a small number of pts in our sample, and more data are needed to support this possibility; we are currently collecting additional data as the number of pts treated is increasing.

In the ALSYMPCA trial, radium-223 was associated with significant lengthening of the time to first skeletal event in pts with secondary bone metastases from mCRPC, which had a positive impact on both quality of life and OS^10,22^. However, concomitant factors such as prolonged androgenic block and/or steroid therapy can render bone more fragile, facilitating osteoporosis and increasing the probability of adverse skeletal events, with subsequent interruption of therapy^23–25^.

For this reason, in pts treated at our centre, two strategies are initiated early in therapy: the first consists of prompt administration of bisphosphonate therapy and supplementation with vitamin D and calcium. The importance of administering zoledronate therapy to pts with mCRPC if they are not already receiving it should be stressed. However, it is not always possible to initiate these therapies at an early stage due to the high risk of mandibular osteonecrosis in some pts. In the phase 3 study, cases of osteonecrosis of the jaw were reported in 0.67% of pts with radium-223 and in 0.33% of those treated with placebo. (Radium-223, Summary of Product Characteristics). Of note, all pts with osteonecrosis of the jaw were also exposed to prior or concomitant bisphosphonates (e.g. zoledronic acid) and prior chemotherapy (e.g. docetaxel). In our cohort, 3 pts showed signs of mandibular necrosis either before or during treatment with radium-223 despite long-term therapy with bisphosphonates; in these pts, there were no signs of toxicity to radium-223 and all cases were treated adequately by surgery and had no additional sequelae.

The second strategy we use in our centre to prevent bone events is the early recognition and treatment of painful metastatic bone lesions with external beam radiotherapy. Eleven of the 63 pts treated with radium-223 received external beam radiotherapy with conventional fractionation with the intent to reduce pain during therapy with radium-223 or in the following months. In all cases, radiation therapy was well tolerated and had no significant short- or long-term adverse effects.

According to data in the literature, a gradual and constant decrease in the levels of ALP is one of the most reliable parameters for evaluation of initial response to treatment^26^. In the pivotal phase 3 trial, radium-223 significantly prolonged the time to an increase in total ALP^10^. In addition, a significantly higher proportion of pts in the radium-223 group had a response according to the total ALP level (≥30% reduction) and normalisation. A correlation between pre-treatment ALP levels (≥146 U/L) and increased risk of death, time to progression, skeletal-related events and bone marrow failure has been noted, which suggests a prognostic value for baseline ALP^27^. For PSA, in the pivotal study a 30% or greater reduction in PSA levels at week 12 was seen in 16% of pts in the radium-223 group vs. 6% of those given placebo^10^. Additional studies have shown a significant correlation between PSA doubling time after two consecutive cycles of therapy and OS and PFS. However, a comparable correlation was not observed between pre-treatment and post-treatment PSA doubling time^28^. In addition, it is possible that pts experience a PSA flare due to release of PSA from lysis of tumour cells^27^. For PSA, there is thus inconclusive evidence to use this as a marker for response, and the available data suggests that the only predictive parameter of efficacy at present is a decrease in ALP during the initial months of therapy^27^. PSA, in association with other parameters such as extent and number of metastases, baseline value of PSA and number of previous chemotherapy lines may, however, be associated with greater OS^29^. In our experience, pts who started early therapy with bisphosphonates had lower values of ALP than those not administered bisphosphonates. However, it is important to emphasise that the majority of our pts started new mCRPC systemic therapy at one month after the end of radium-223 treatment, which was a potential confounder for evaluation of PSA, ALP and disease response in the next months of follow-up. In addition, similar to other reports^30–33^, pts who had previously been administered abiraterone (35 of 63 pts) did not show evidence of a significant increase in the incidence of skeletal events or relevant toxicity.

As reported in the literature and in our experience, therapy with radium-223 leads to improvement in bone pain^10,27,34^, which favoured dose reduction or discontinuation of pain medication in 12 of our 63 pts; in 38 pts, additional pain medication or dose increase was not needed, while adequate pain control was not achieved in only 8 pts and intensification of pain therapy was required. In the ALYSMPCA trial, 2% of pts in the radium-223 group and 1% of pts in the placebo group had no pain or analgesic use at baseline, and radium-223 treatment favoured less use of opioids compared to placebo^10^. In general, by analysing the different groups at baseline, considering the third and sixth cycles of radium-223, NRS scores showed a significant decrease over time during therapy. Moreover, 67% of pts were able to complete all six administrations of radium-223, which in our opinion was very good in consideration of the advanced stage of disease treated. Avoidance of the general health deterioration allows the patient to complete all radium-223 courses and thus to maximizes the chances of survival; in the analysis of Huynnh-Le at al., pts experiencing AEs during treatment received only one-half of the prescription-dose with a negative impact on survival^35^. Furthermore, in our sample, 42% and 28% of pts had, respectively, a PR and SD at one month after the end of treatment.

The most important limits of this study are its retrospective nature, the small sample size, and the lack of data regarding anaemia and other markers since this analysis was made during the initial phase of recruitment of pts, as well as the lack of analysis on potential confounders for evaluation of disease response at one month after treatment and trends in crucial parameters such as PSA and ALP. In the literature, there is a lack of clinical evidence about the correct management of pts treated with radium-223; our study stresses the need for early identification and correct management of clinical variables that can hamper the completion of radium-223 administrations, in order to obtain the maximum effectiveness from the treatment.

## Conclusions

In our cohort, median survival was 15 months, with a median OS of 23 months for pts who underwent radium-223 as first line therapy. For these reasons, even if the new regulatory indications in Italy recommend radium-223 treatment only in third line, we strongly suggest the possibility to consider first- or second-line radium-223 in pts who are ineligible due to comorbidities. Moreover, these encouraging results were likely to early recognition and treatment of the main factors responsible for potential failure and/or early interruption of therapy with radium-223. The availability of radium-223 has undoubtedly changed the treatment scenario for management of mCRPC. In our experience, radium-223 can be considered well tolerated and effective, and confirm the favourable results reported in the literature. We endeavour to collect more data in the future regarding anaemia, the use of bone-targeting agents and the role of external beam radiotherapy in the treatment of bone lesions, to confirm with statistical significance the hypothesis we have formulated.

## Data Availability

The datasets used and/or analyzed during the current study are available from the corresponding author on reasonable request.
